# Artificial high birefringence in all-dielectric gradient grating for broadband terahertz waves

**DOI:** 10.1038/srep38562

**Published:** 2016-12-09

**Authors:** Meng Chen, Fei Fan, Shi-Tong Xu, Sheng-Jiang Chang

**Affiliations:** 1 Institute of Modern Optics, Nankai University, Key Laboratory of Optical Information Science and Technology, Ministry of Education, Tianjin 300071, China; 2 Tianjin Key Laboratory of Optoelectronic Sensor and Sensing Network Technology, Tianjin 300071, China

## Abstract

Subwavelength dielectric gratings are widely applied in the phase and polarization manipulation of light. However, the dispersion of the normal dielectric gratings is not flat while their birefringences are not enough in the THz regime. In this paper, we have fabricated two all-dielectric gratings with gradient grids in the THz regime, of which artificial birefringence is much larger than that of the equal-grid dielectric grating demonstrated by both experiments and simulations. The transmission and dispersion characteristics are also improved since the gradient grids break the periodicity of grating lattices as a chirp feature. From 0.6–1.4 THz, a broadband birefringence reaches 0.35 with a low dispersion and good linearity of phase shift, and the maximum phase shift is 1.4π. Furthermore, these gradient gratings are applied as half-wave plates and realize a linear polarization conversion with a conversion rate over 99%, also much higher than the equal-grid gratings. These gradient gratings show great advantages compared to the periodic gratings and provide a new way in the designing of artificial birefringence material.

Terahertz (THz) wave, one of the least-explored areas in the electromagnetic (EM) spectrum, has attracted a great deal of research attentions since the development of efficient THz sources and sensitive detectors recently. It shows great application prospect in communication^1^, imaging^2^, and spectroscopy^3^. To realize these applications, THz functional devices such as modulator^4^, filter^5^, absorber^6^ and sensor^7^ are indispensable. Phase and polarization are two basic properties of EM waves. They can not only carry valuable information but also control the light propagation^8^. Accordingly, the phase and polarization devices such as phase shifter^9^, wave retarder^10^, polarizer^11^ and polarization converter^12^ are of great importance. Conventional methods for the phase and polarization controlling depend on the properties of natural birefringence materials, which support different phase delays along the two orthogonal optical axes. These natural birefringence materials are very limited in the THz regime due to the low birefringence, large loss, bulk in size, and high price^13^,^14^,^15^. For instance, the typical liquid crystal E7 has its birefringence of only 0.12 but a large loss of about 1 cm^−1^ at 1 THz^16^; the ordinary and extraordinary refractive indices of crystalline quartz are 2.109 and 2.155 at 1 THz respectively, so the thickness of its quarter wave plate is over mm thickness in the THz regime^17^, which is determined by *d* = *πc*/2*ω*Δ*n*, where *c* is the speed of light in vacuum, *ω* is the angular frequency of light, Δ*n* is birefringence coefficient of materials.

Therefore, many artificial birefringence materials have been proposed to solve these problems. Based on artificial electromagnetic structures, such as surface plasmon, metamaterials, photonic crystals, and subwavelength grating, the spatial asymmetry of the structure can get high anisotropy to obtain THz phase shifters, polarization converters, and one-way transmission^18^,^19^,^20^,^21^,^22^,^23^,^24^,^25^,^26^,^27^,^28^,^29^,^30^. Zhu *et al*. proposed a THz polarization conversion with a hyperbolic metamaterial waveguide^18^, while Wei. *et al*. achieved controllable polarization conversion in microwave region with similar multilayer metamaterial^19^, but these multilayer stacking structures were rather difficult in fabrication^20^,^21^,^22^. Liu *et al*. proposed a single-layer birefringence metasurface as a quarter-wave plate at 0.91~1.45 THz^23^, and Wang *et al*. proposed a switchable quarter wave plate by using the phase transition property of VO_2_^24^. However, these plasmonic structures have the low transmittance due to their ohmic loss^25^,^26^,^27^ or operate as the reflective devices^28^,^29^,^30^.

All-dielectric artificial birefringence structures can overcome the above difficulties. High transmission is expected since they are completely made of dielectric materials, and the thin thickness of device can also limit the absorption loss. Subwavelength dielectric gratings show great advantages to control the phase and polarization of the EM waves, which have different effective refractive index perpendicular and parallel to the grating grids respectively. Many devices for phase and polarization manipulations based on these subwavelength gratings have been proposed in the visible, near infrared and THz band^31^,^32^,^33^,^34^. However, these investigations mainly focused on the conventional dielectric gratings which have a constant grating period. The birefringence of this equal-periodic grid grating is still limited and the phase difference of π is difficult to be obtained, so they only can be used as a quarter-wave plate but not a half-wave plate.

In this letter, we have fabricated two all-dielectric gradient gratings with a series of gradient grating grids for artificial high birefringence in the THz regime. The gradient grids significantly increase the birefringence coefficient and improved the transmission and dispersion of the device, compared to the normal dielectric gratings. The birefringence in the range of 0.6–1.5 THz reaches 0.35 with the maximum phase difference of 1.4π. To further demonstrate their applications in polarization conversion, these devices have been experimentally confirmed as a half-wave wave plate with a linear polarization conversion rate of over 99%.

## Results

**Figure 1:**
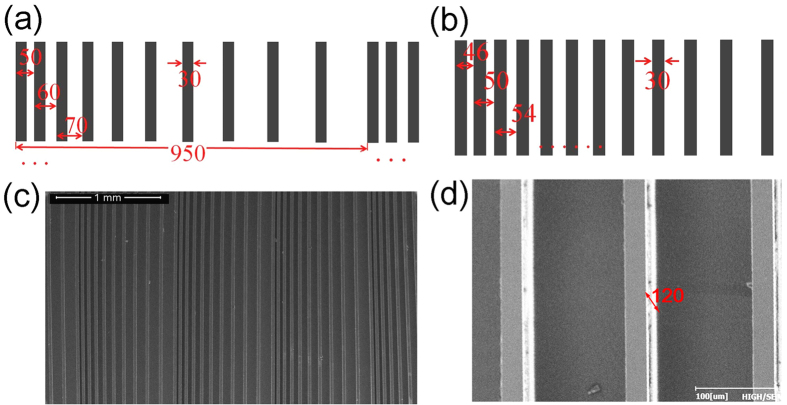
(**a**) Schematic of the PGG with a gradient step of 10 μm and the period of 950 μm. (**b**) The MGG with a gradient step of 4 μm without periodicity; (**c**) SEM image of the PGG; (**d**) Oblique view of SEM image of MGG. The height of grating ridge is 120 μm.

Two dielectric gratings have been fabricated by MEMs fabrication: one is called as periodic gradient grating (PGG), and the other is monotonic gradient grating (MGG). The details for device fabrication can be found in the **Method Section**. By lithography and etching, relief grating structures are obtained on the high resistance silicon wafers of 10 KΩ cm, where the grating ridges and grooves are alternatively arranged as shown in Fig. 1. A pair of grating ridge and groove forms a grating grid. All the groove depth is 120 μm as shown in Fig. 1(d), and all the ridge width is 30 μm as a constant. For PGG as shown in Fig. 1(a) and (c), 10 gradient increasing grids line as a gradient grid group, which has an initial grid width of 50 μm and a uniform difference of 10 μm in the adjacent grids, so the gradient grid group has 950 μm width. Several gradient grid groups are periodically arranged to form the grating PGG. Therefore, this PGG is still a periodic structure, but a series of aperiodic gradient grids formed one large lattice cell. For MGG structure as shown in Fig. 1(b), monotonically gradient increasing grids form the whole grating without any periodicity. The difference is 4 μm in the adjacent grids, and the initial period is 46 μm. Therefore, the pure linear-chirp breaks the periodicity in the MGG.

**Figure 2:**
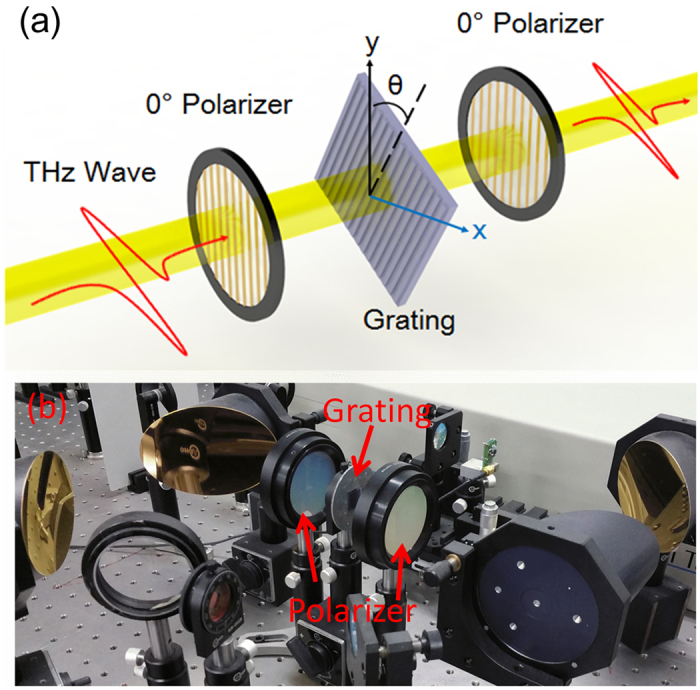
(**a**) Schematic of the birefringence measuring experiment configuration; (**b**) Photo of the THz-TDS system and birefringence measuring experiment components.

Analogous to the uniaxial crystals, we define the optical axis of the dielectric gratings as the direction paralleled to the grating ridge. To study the birefringence of the dielectric gratings, the THz time domain pulse of the different polarization directions have been measured by a four parabolic mirror THz time domain spectroscopy (THz-TDS) system, as shown in Fig. 2(b). The dielectric grating is placed at the co-focus position of the THz-TDS system. Two polarizers with the same polarizing direction (here along the *y* axis) are inserted before and after the grating chip, to ensure that the incident and detected polarizations are consistent. The grating chip can be rotated with an angle *θ* between the optical axis and the polarizing direction of the polarizer, as shown in Fig. 2(a). THz pulses are generated by a low-temperature grown GaAs photo- conductive antenna with a 50 μm slit, and detected by a ZnTe crystal. All the experiments are carried out at room temperature with the humidity lower than 5%.

The incident wave after the first polarizer can be expressed as 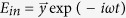. Here 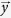 is the unit vector along *y* axis and *ω* is the angular frequency. As shown in Fig. 2(a), *θ* = 0° is the TE mode for the grating structure, and *θ* = 90° is the TM mode. Then the wave of arbitrary *θ* passing through the grating is


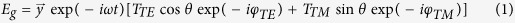


where *φ*_*TE*_ and *φ*_*TM*_ are the phases, *T*_*TE*_ and *T*_*TM*_ are the amplitude transmissions of the TE and TM mode. After the second polarizer, the output amplitude signal of arbitrary *θ* can be expressed theoretically by


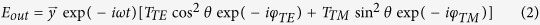


**Figure 3:**
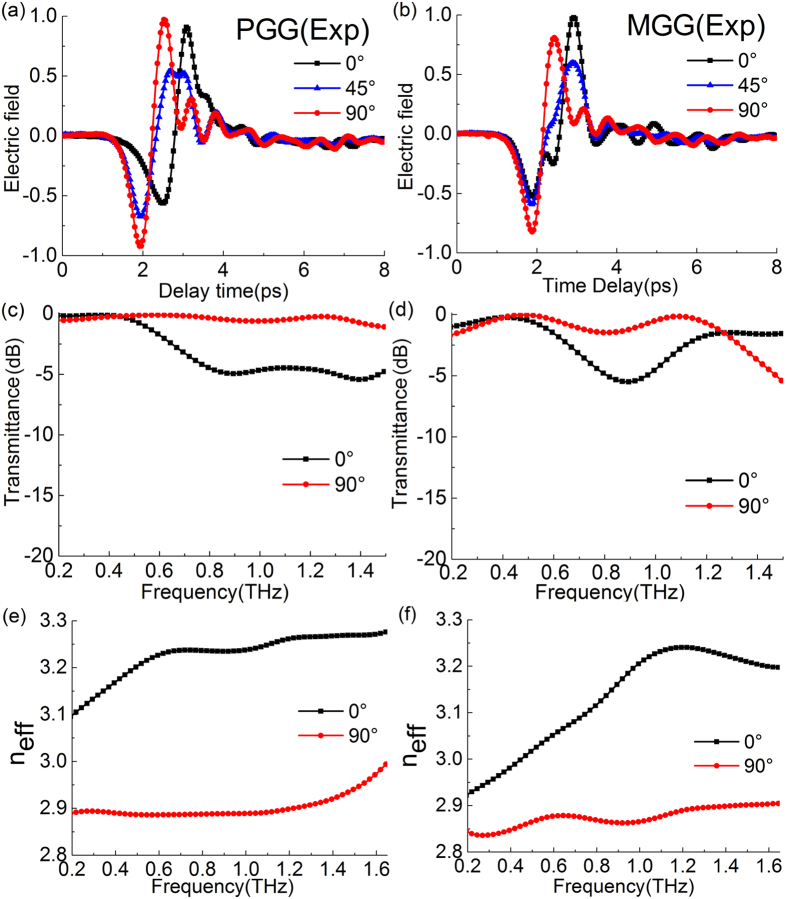
Experimental time domain pulses with different polarization angles of (**a**) PGG and (**b**) MGG. Experimental transmission spectra of TE (0°) and TM (90°) polarization modes obtained by Fourier transform of the time domain data for (**c**) PGG and (**d**) MGG. Experimental effective refractive indexes of TE (0°) and TM (90°) modes obtained by Fourier transform of the time domain data and Eq. 8 for (**e**) PGG and (**f**) MGG.

The experimental time domain pulse signals with *θ* = 0°, 45° and 90° of PGG are shown in Fig. 3(a), which indicates that as the *θ* increases, the pulse delay becomes smaller and the pulse peak moves forward. The positive pulse peak locates at 3 ps for TE mode, while this peak moves forward to 2.5 ps for TM mode, so the phase shift of TM wave is smaller than that of TE wave. Figure 3(b) shows the results of MGG in a similar trend as the PGG. Therefore, a large birefringence effect of both PGG and MGG can be directly seen from the THz-TDS data.

The power transmission spectra are obtained by Fourier transform of the measured time domain data. The details for **data processing** can be found in the **Method Section**. In general, the flat and high transmission is expected in the broadband specrum. The gradient gratings break the space periodicity, avoid the guided mode resonance^35^,^36^ and cause the continuous gradient in the space phase distribution. As shown in Fig. 3(c) and (d), the transmission spectra of the two gradient gratings are relatively smooth and transparent without strong resonance dips.

To further assess the birefringence properties of these gradient gratings, their effective refractive indexes are calculated. The results of the PGG is shown in Fig. 3(e). In 0.4–1.4 THz, the dispersion of the TE and TM modes are both small. The effective refractive index of the TE mode is 3.25, while the TM mode takes its value of 2.9. Therefore, the birefringence of larger than 0.3 is achieved in a board bandwidth of 1.1 THz for the PGG. By comparison with the MGG shown in Fig. 3(f), the TE mode of the MGG shows a significately positive dispersion below 1.05 THz. Its birefringence also reaches up to 0.35 at 1.05 THz, but its bandwidth is much narrower than that of the PGG.

**Figure 4:**
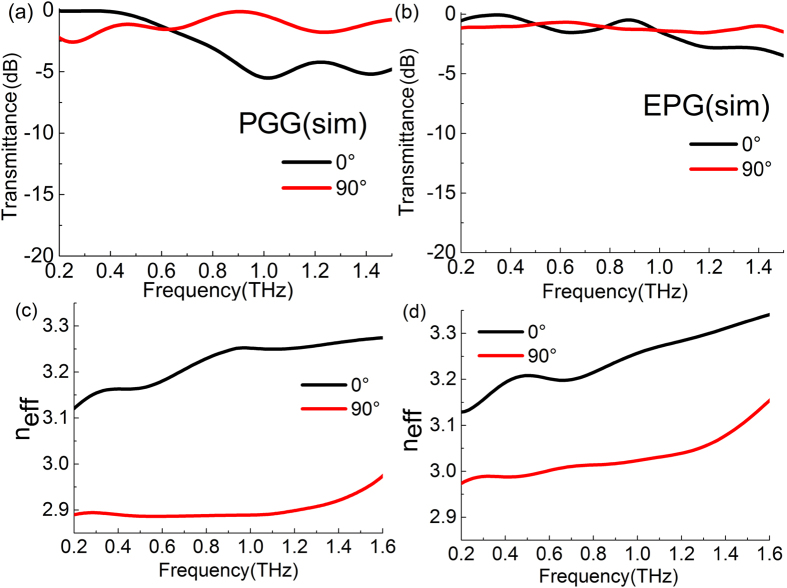
Simulative transmission spectra of TE (0°) and TM (90°) polarization modes for (**a**) PGG and (**b**) EPG; Simulative effective refractive indexes of TE and TM polarization modes for (**c**) PGG and (**d**) EPG.

The transmission and phase characteristics of the PGG are numerically simulated by the CST software, as shown in Fig. 4(a) and (c). The simulation results fit well with the experiment data. Although the PGG has a long period of 950 μm, the gradient effect of the grids is more significant than the diffraction effect of the long path periodicity. According to the simulation, we find that the main energy of the transmitted wave is in the zero order diffraction and the higher orders can be neglected. An equal-periodic grid grating (EPG) is also modeled by CST simulation in Fig. 4(b) and (d) for further comparision. The grid period of the EPG is 95 μm and its grating ridge width is 30 μm. These parameters are selected to make that the filling factor of the EPG (*f*_EPG_ = 30/95) equals to the PGG (*f*_PGG_ = 30 × 10/950). For the grating of which period is far smaller than the wavelength of incident light, its effective refractive index can be calculated by the effective medium theory^37^. According to this theory, the effective refractive indexes of the gratings with the same filling factors should be equal. But it is not applicable for our subwavelength grating since their periods is in the same order with the THz wavelength. For these dielectirc subwavelength microstrctures, their airtifical birefringence is determined mainly on the four factors: the refractive index difference and filling factor between constituent materials, the height of microstrcture units (that is dependent on the etching depth), spatial asymmetry of structure, the spatial distribution relation among microstrcture units. As shown in Fig. 4(b), the effective refractive indexes of the PGG and EPG are quite different because their grids have the different spatial distribution relations though other three factors are the same. The difference of popagation constant between TE and TM modes can be expressed as follows:


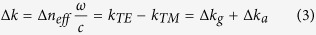


where *∆k*_*g*_ is the difference of popagation constant between TE and TM modes from the spatial asymmetry of the grating grid unit, and *∆k*_*a*_is the additional wave vector introduced by the gradient distribution of the grid units.

The gradient change of the grating grids generates the gradient distribution of spacial phase in the TM mode for PGG and MGG, and these gradient spacial phases introduce an additional wave vector *∆k*_*a*_, which reduce the effective refractive index of TM mode. Of course, this structure also influences the TE mode but mainly on TM mode, which result that the airtifical birefringence of the gradient grating is larger than that of the normal grating. Essentially, this structure further breaks the symmetry of the two orthogonal directions in the *X-Y* plane, and also damage the periodicity of the normal grating to eliminate grating diffraction and resonance effects, so the bandwidth and dispersion are also improved as a chirp grating feature.

**Figure 5:**
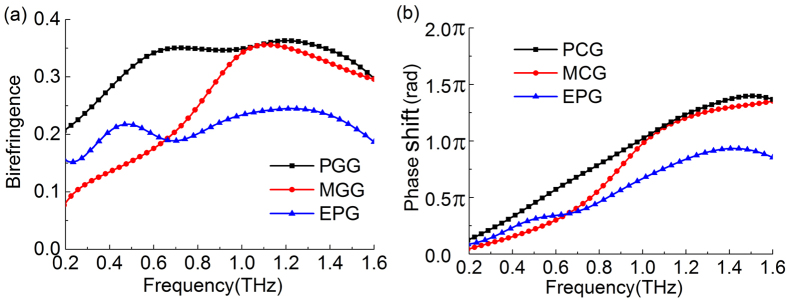
(**a**) The birefringence coefficient Δ*n* and (**b**) the phase shift Δ*φ* of the PGG, MGG and EPG in the THz spectrum.

Figure 5(a) compares the birefringence coefficients of the PGG, MGG and EPG in the THz regime. The PGG has a high birefringence in the whole spectrum range in Fig. 5(a), and its birefringence coefficient reaches up to 0.35 in 0.6–1.4 THz. The birefringence coefficient of MGG is very small initially in the low frequency, only 0.08 at 0.2 THz, but it greatly increases to a peak value of 0.35 at 1.1 THz, and then it drops down at a higher frequency. The birefringence coefficients of these two gradient gratings are much larger than that of the EPG. The birefringence coefficient of the EPG is only about 0.15–0.2 in the whole spectrum range shown in Fig. 5(a). We notice that S. Kruk *et al*. have reported a dielectric metasurface that its artificial birefringence reached 0.3 to realize polarization conversion in the 1550 nm band^38^. The basic principle of these two works is the same that the device controls specific spatial phase distribution by using the gradient changes of its subwavelength structure units, so as to realize large artificial birefringence and polarization conversion effectively. Both works prove that the gradient subwavelength structures are superior to the simple periodic subwavelength structures for the artificial birefringence and polarization control when other impact factors are all the same. In a sense, our gradient grating structure can be also seen as a metasurface, but S. Kruk *et al*.’s structure is much more complicated than ours.

The phase shifts of the two orthogonal polarization states in these three gratings are shown in Fig. 5(b), expressed by Δ*φ* = *φ*_*TE*_−*φ*_*TM*_. All the three phase shifts are increased with the frequency. For the same thickness, a larger birefringence leads to a larger phase shift, so the phase shift of the PGG and MGG reach to the maximun value of 1.4π at 1.5 THz. Thus, they not only can be used as a quarter-wave plate at low frequency, but also a half-wave plate at high frequency. Moreover, the PGG indicates a good linear-phase shift characteristic in the with frequency range of 0.2–1.4 THz, which can be used as a broadband THz linear phase shifter. The maximun phase shift of the EPG is only 0.9π at 1.4 THz, and it is not linearly increased with its value of 0.8~0.9π in 1.2~1.6 THz. Therefore, compared to the EPG, the gradient gratings can realize a larger phase shift. They can obtain enough phase shift with a smaller thickness so that they can reduce the material loss.

## Discussion

**Figure 6:**
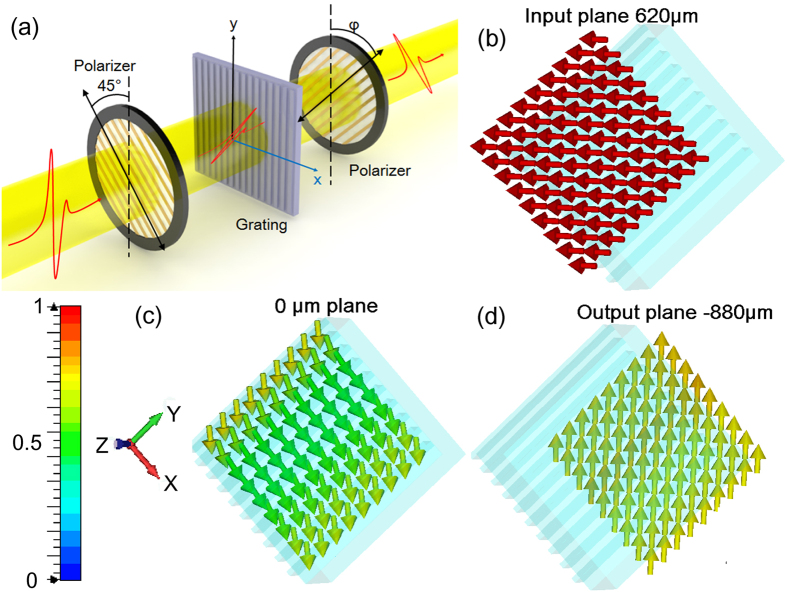
(**a**) Schematic of the polarization conversion experiment configuration. (**b–d**) The distribution of the electic vectors at (**b**) the input plane (**c**) inside the dielectric grating and (**d**) at the output plane simluated with PGG at 1.05 THz. The arrows indicate the direction of the electic field vectors.

Furthermore, we discuss the application of these all-dielectric gradient gratings in linear polarization conversion as a half wave plate. The experiment configuration is performed in the THz-TDS system as shown in Fig. 6(a). A polarizer is placed before the gradient grating with a fixed angle of 45° between its polarizing direction and the optical axis of the grating. Thus only the 45° component of the THz wave is incident on the grating and the initial amplitudes of the TE and TM mode are the same. In the ideal case, when the transmittances of the TE mode and TM mode are equal and the phase difference between TE and TM is π, the polarization state of the THz wave through the grating will be rotated from 45° to −45°. The electirc field distributions in this model are also numberically simulated by CST software, as shown in Fig. 6(b)−(d). A plane wave of 1.05 THz with a horizontal linear polarization state is incident into the 45° PGG as shown in Fig. 6(b). Then the polarization direction changes inside the grating. The electirc vectors are rotating clockwise in 0 μm plane (i.e. the middle cutting plane of PPG) as shown in Fig. 6(c), which change to be a right-handed elliptically polarized light. After passing through the grating, the electirc vectors become vertical at the output plane, so the polarization state of the wave is rotated 90° perfectly.

To assess the linear polarization conversion rate of these gratings, the converted component and unconverted component should be distinguished in the detections. Another polarizer is set behind the gratings with an angle *α* between its polarizing direction and the optical axis. Then the amplitude of the output THz wave can be calculated by:


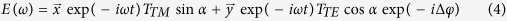


where *T*_*TE*_ and *T*_*TM*_ are the amplitude transmission of TE and TM modes through the gratings, which can be found in Fig. 3(c) and (d). Thus the out power spectrum is expressed as:


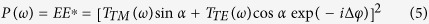


**Figure 7:**
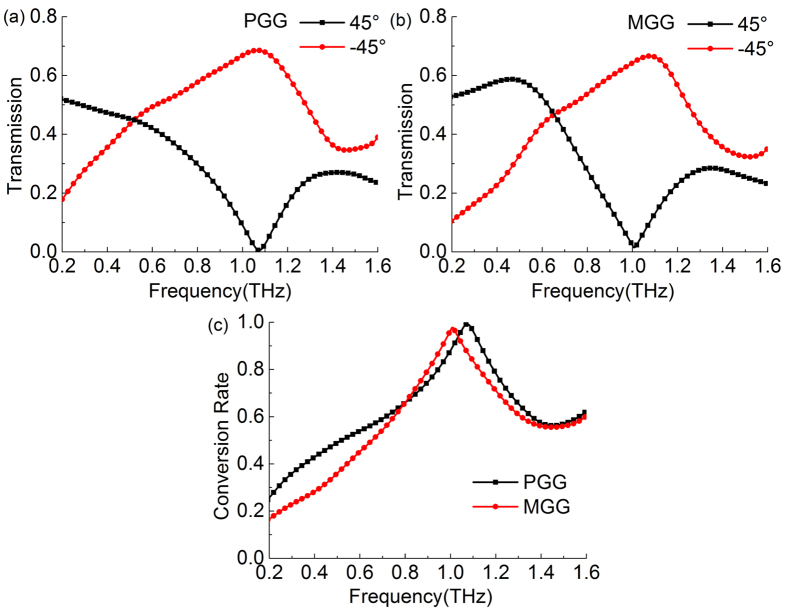
Experimental transmission spectra of converted (−45°) and unconverted components (45°) for the polarization conversion configuration by using (**a**) PGG and (**b**) MGG. (**c**) Experimental polarization conversion rate of PGG and MGG in the THz frequency range.

At the working frequency of a half waveplate, the phase shift Δφ = π. When α = −45°, the converted component can pass through the second polarizer with a high transmitance according to Eq. (5). Conversely, the unconverted component is obtained when α = 45°. In theory, the two components should be complementary in addtion to insertion loss. Here we focus on the polarization conversion effect of the gratings, and the conversion rate is defined as


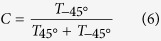


where *T*_−45°_ and *T*_45°_ are the amplitude tranmission for the converted component and unconverted component, respectively. Figure 7(a) shows the amplitude transmission spectra of the PGG in the experiments. At 1.06 THz, the transmitance of converted component of the PGG reaches 69%, and the unconverted component is only 1%. Its maximum conversion rate is 99.3% at 1.06 THz, as shown in Fig. 7(c). At higher frequencies, the conversion rate decreases since the birefringence of the PGG is large, and the phase shift is linearly increased far away from π as shown in Fig. 5(b). Moreover, we should note that this best conversion frequency 1.06 THz has a small deviation compared to Δ*φ* = π at 0.98 THz shown in Fig. 5(b). This linear polarization conversion is both determined by the value of phase shift and the tranmission of TE and TM modes. Only when *T*_*TE*_ = *T*_*TM*_ and Δ*φ* = π at a certain frequency, the output wave should be a strict −45° linearly polarized light after the grating. Therefore, the output is not strictly −45° polarized light since *T*_*TE*_ ≠ *T*_*TM*_ in our case, and the best conversion frequency will deviate from the frequency of Δ*φ* = π. As a result, the conversion rate cannot reach 100%. The transmission spectra of the MGG are shown in Fig. 7(b). Its conversion rate is slightly smaller than PGG, which is 97.7% at 1 THz.

**Figure 8:**
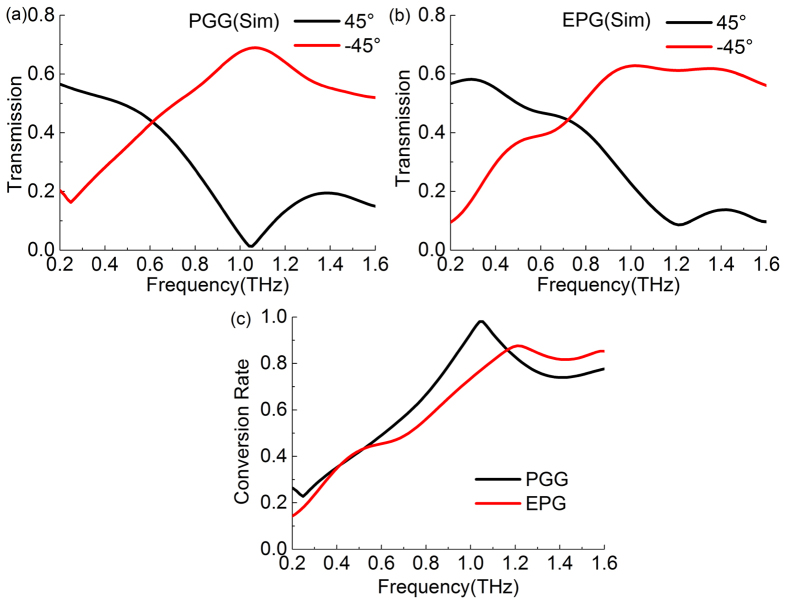
Simulative transmission spectra of converted (−45°) and unconverted (45°) components for the polarization conversion configuration by using (**a**) PGG and (**b**) EPG. (**c**) Simulative polarization conversion rate of PGG and EPG in the THz frequency range.

These polarization conversion models for the PGG and EPG are also simulated by the CST software. The result of the PGG is shown in Fig. 8(a) with the maximum transmitance of 68.9% at 1.07 THz in a good agreement with the experiment data in Fig. 7(a). The simulation result of the EPG is given in Fig. 8(b) as a comparision. Its maximum conversion rate 87.4% is located at 1.21 THz, which is much lower than the maximum conversion rate of the PGG. As shown in Fig. 5(b), the phase shift of EPG cannot reach π, but it has a peak value of 0.9π at 1.4 THz, so it is not linearly increased with its value of 0.8~0.9π in 1.2~1.6 THz. This is the reason that the maximum conversion rate of EPG is small than that of the PGG. In spite of this, in the frequency band of 1.2~1.6 THz, its conversion rate is larger than that of the PGG because its phase shift remains 0.8~0.9π in this band. These results are in good agreement with Fig. 5(b). Therefore, the PGG has the highest polarization conversion rate among the three gratings, and this gradient grating has a larger birefringence, boarder bandwidth, lower dispersion, and better linearty of phase shift than those of the two other gratings, especially the normal dielectric grating.

## Conclusion

In conclusion, two all-dielectric gradient gratings with gradient grids are fabricated for artificial high birefringence in the THz regime. The experiments and simulations show that the gradient grating grids effectively increase the birefringence compared to the normal gratings, which is contributed by the additional popagation constant introduced by the gradient distribution of the grid units. Moreover, the bandwidth and dispersion are also improved due to breaking the periodicity of the grating as a chirp feature. A flat birefringence of larger than 0.35 is achieved from 0.6 to 1.5 THz and the maximum phase shift reaches 1.4π. A polarization conversion experiment is performed by using the gratings as half-wave plates, which shows the conversion rate of PGG is larger than 99%. Therefore, these gradient gratings with large birefringence, high transmission, and low loss have great applications in the control of the phase and polarization of THz wave.

## Method

### Fabrication

The gratings used in our experiments are fabricated by MEMs technology^39^,^40^. A 500 μm thickness Si wafer with a high resistivity of 10 kΩ cm is cleaned and a 5 μm layer of photoresist is spun onto the wafer. Then, the wafer is exposed by UV light through a mask to yield the expected structure, and is shaped by the inductively coupled plasma etching. The etched depth is controlled by the time of etching for 40 min, and finally the etched depth is 120 μm measured by a step profiler. After cleaning out the photoresist and dicing the wafer with laser, the grating samples with the size of 1 cm × 1 cm are obtained.

### Data processing

The data in Fig. 3(a) and 3(b) are directly collected from the experiments. These experimental data can be indicated as *E*_*g*_(*t*). Here an air signal is used as reference. Its transmission signal *E*_*air*_(*t*) is measured but not shown in the figures. Then their amplitudes in the frequency domain are obtained by Fourier transform of the time domain pulses. The amplitude spectrum of the gratings are *E*_*g*_(*ω*), and the reference is *E*_*air*_(*ω*). Therefore, the transmission spectra shown in Fig. 3(c) and (d) are calculated by^39^,^40^,^41^


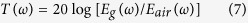


To calculate the effective refractive index of the gratings, the phase specra are also acquired by the Fourier transform of the time domain data. *φ*_*g*_(*ω*) (that is *φ*_*TE*_(*ω*) or *φ*_*TM*_(*ω*)) is the phase specrum of grating sample, and *φ*_*air*_(*ω*) is that of air reference. The effective refractive index can be calculated by^39^,^40^,^41^


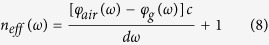


where *d* = 500 μm is the thickness of the samples. These results of effective refractive indexes are shown in Figs 3 and 4.

The birefringence coefficient Δ*n* and the phase shift Δ*φ* between TE and TM modes shown in Fig. 5 are expressed as follows


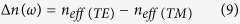



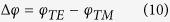


### Numerical simulation

In this letter, all the numerical simulation are taken by the CST software. For the PGG, a large period of 950 μm is ploted to bulid the model, while the EPG is modeled with its single period of one groove and ridge. The silicon is set to be lossless with its permittivity of 11.7. The plane wave is set as the source before the grating. Nonlinear mesh is used here with its minimum size of 1 μm. Two pairs of the periodic boundary condition are set at both *x* and *y* directions. In the numerical simulation, the periodic boundary condition can greatly decrease the model size, and thus reduce the requirement of the computer memory. Due to the lack of the periodicity, the simulation model of the MGG needs very large memory beyond our computational capability, so its simulation result is absent in this paper.

## Additional Information

**How to cite this article**: Chen, M. *et al*. Artificial high birefringence in all-dielectric gradient grating for broadband terahertz waves. *Sci. Rep.* **6**, 38562; doi: 10.1038/srep38562 (2016).

**Publisher's note:** Springer Nature remains neutral with regard to jurisdictional claims in published maps and institutional affiliations.
